# Diagnostic challenge of Creutzfeldt-Jakob disease in a patient with multimorbidity: a case-report

**DOI:** 10.1186/s12883-023-03401-5

**Published:** 2023-10-02

**Authors:** Amber Yaqub, Mohammad Kamran Ikram, Jeroen Blankevoort, Mohammad Arfan Ikram

**Affiliations:** 1 Department of Epidemiology, Erasmus University Medical Center, Rotterdam, the Netherlands; 2 Department of Neurology, Erasmus University Medical Center, Rotterdam, the Netherlands; 3 Department of Neurology, Flevoziekenhuis, Almere, the Netherlands

**Keywords:** Creutzfeldt-Jakob disease, Situs inversus totalis, Churg Strauss syndrome, Multimorbidity, Rare diseases, Case report

## Abstract

**Background:**

Creutzfeldt-Jakob disease (CJD) is a rapidly progressive and ultimately fatal neurodegenerative condition caused by prions. The clinical symptoms of CJD vary with its subtype, and may include dementia, visual hallucinations, myoclonus, ataxia, (extra)pyramidal signs and akinetic mutism. In the early course of disease however, several clinical symptoms of CJD may mimic those of co-existing morbidities.

**Case presentation:**

We report a male in his 60s with a history of situs inversus totalis and Churg Strauss syndrome, who presented with speech fluency disturbances, neuropsychiatric symptoms and allodynia, a few months after becoming a widower. Initially presumed a bereavement disorder along with a flare-up of Churg Strauss, his symptoms gradually worsened with apraxia, myoclonic jerks and eventually, akinetic mutism. MRI revealed hyperintensities at the caudate nucleus and thalami, while the cerebrospinal fluid was positive for the 14-3-3 protein and the real-time quick test, making the diagnosis of CJD highly probable. This case illustrates the complexities that may arise in diagnosing CJD when pre-existing multimorbidity may cloud the clinical presentation. We also discuss the potential mechanisms underlying the co-occurrence of three rare conditions (situs inversus totalis, Churg Strauss syndrome, CJD) in one patient, taking into consideration the possibility of coincidence as well as common underlying factors.

**Conclusions:**

The diagnosis of CJD may be easily missed when its clinical symptoms are obscured by those of pre-existing (rare) multimorbidity. This case highlights that when the multimorbidity has neurological manifestations, an extensive evaluation remains crucial to establish the diagnosis, minimize the risk of prion-transmission and provide appropriate guidance to patients and their caregivers.

**Supplementary Information:**

The online version contains supplementary material available at 10.1186/s12883-023-03401-5.

## Background

Creutzfeldt-Jakob disease (CJD) is a rare, rapidly progressive and invariably fatal neurodegenerative disorder caused by prions. Prions are misfolded proteins, formed by conversion of the normal cellular prion protein with an alpha-helical structure (PrP^c^) to a β-pleated scrapie (PrP^Sc^) form, that predispose to spongiform encephalopathy [1]. Based on its origin, four types of CJD are currently distinguished: sporadic, familial, iatrogenic, and the variant form. Sporadic CJD (sCJD) accounts for 85–90% of all CJD cases and manifests when normal prion protein spontaneously misfolds into PrP^Sc^, typically affecting individuals of about 65 years old [2]. Familial CJD is inherited and results from mutations in the prion protein gene (PRNP), accounting for 5–10% of all cases, with an earlier age of onset of around 40–50 years [3]. Variant CJD (vCJD), which is related to bovine spongiform encephalopathy (also known as ‘mad cow disease’), is caused by consuming meat products contaminated with prions, affecting less than 1% of all CJD cases [4]. Iatrogenic CJD, also affecting less than 1% of cases, is related to exposure to a known source, such as contaminated medical instruments or growth hormone injections [5]. The annual incidence of CJD is about 1–2 cases per million people, and surveillance programs are active worldwide to monitor the disease, [6], [7] according to well-defined criteria with high sensitivity (92.2%) [8]. Although the clinical symptoms of CJD are known to vary among subtypes, they generally include a rapid decline in cognitive function, motor symptoms, myoclonus, and eventually akinetic mutism. In the early course of disease however, cardinal features of CJD may be obscured by those of pre-existing multimorbidity, which can result in a diagnostic delay that carries a risk of transmission. To illustrate the above, we report a case of a man in his 60s, with a history of situs inversus totalis and Churg Strauss syndrome who had an atypical presentation of CJD. His multimorbidity complicated the diagnosis, and posed a diagnostic challenge between the sporadic and variant form of the disease. We discuss the neurologic examination, laboratory assessments and radiologic findings, along with a discussion of potential mechanisms underlying the co-occurrence of these three conditions in the same patient.

### Case presentation

In early 2020, the Dutch National Surveillance Center for Creutzfeldt-Jakob Disease (CJD) received a report from the chief neurologist of a previously independent, 63-year-old Asian male. The patient was referred to the neurology outpatient clinic due to suspected dementia, following a 1-year history of disturbances in speech fluency, memory loss, and behavioral changes. The issues in speech fluency started at the end of 2018, shortly after the patient’s spouse had passed away and were initially believed to be part of a bereavement disorder. The patient used to be proficient in both Dutch and his mother tongue, but over the course of 2019, he experienced difficulty recalling words and started stuttering in both languages. According to his daughter, he also experienced problems in short- and long-term memory, with increasingly worsening anger outbursts.

**Fig. 1:**
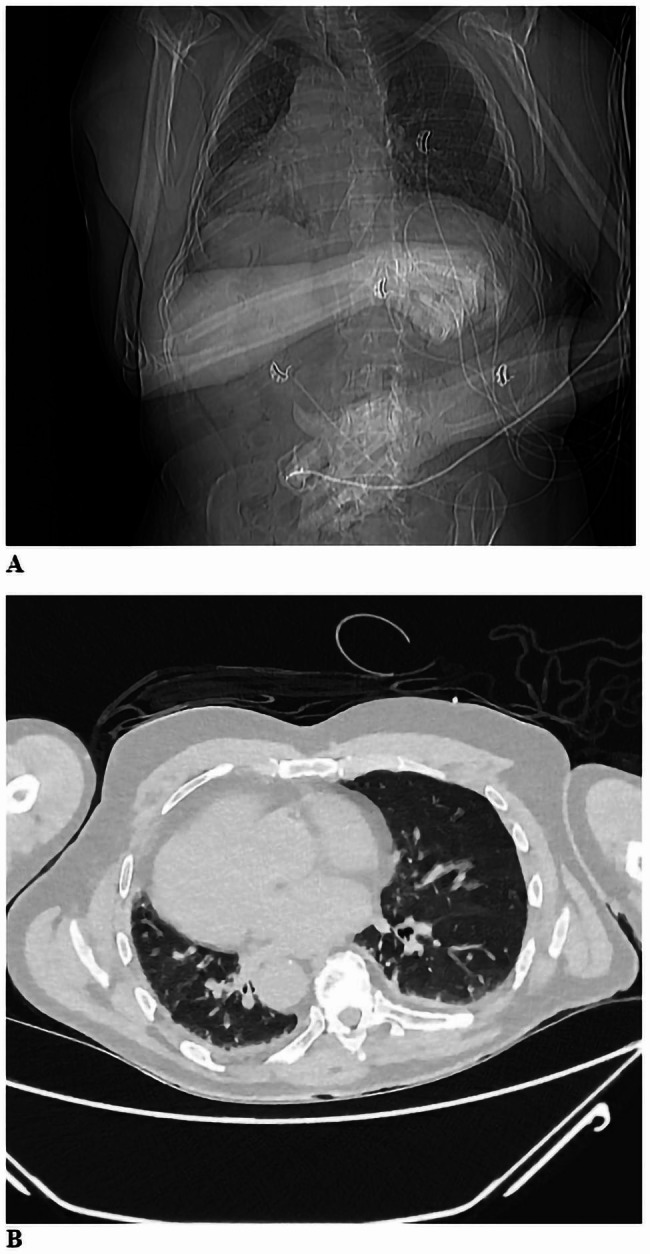
Situs inversus totalis. Situs inversus totalis on X-ray **(A)** and CT **(B)**

The patients’ medical history consisted of situs inversus totalis (Fig. 1) and eosinophilic granulomatosis with polyangiitis (EGPA), better known as Churg Strauss syndrome (CSS). The diagnosis of CSS was established in 1998, based on a combination of clinical criteria, including asthma, peripheral blood eosinophilia, systemic vasculitis of the medium and small vessels, glomerulonephritis and mononeuritis multiplex. Blood tests, including antineutrophil cytoplasmic antibodies (p-ANCA and c-ANCA) supported the diagnosis. Extended details of the patient’s diagnosis of CSS are noted in Supplementary File 1. There was no history of surgery, blood transfusions, hormonal injections, or relevant travel history after 1980, and no family history of dementia or prion disease. The prescribed drugs included amitriptyline, pregabalin, diuretics and prednisone. Concurrent to the neuropsychiatric symptoms mentioned earlier, the patient had been experiencing worsening allodynia, which was attributed to small fiber neuropathy as a part of CSS. Accordingly, the dosage of amitriptyline and pregabalin was increased in an attempt to alleviate the symptoms, but without the desired effect. As the patient’s stuttering, memory problems, anger outbursts, and allodynia continued to worsen despite changes to his treatment regimen for CSS throughout 2019, he was eventually referred to the neurology outpatient clinic for suspected dementia.

### Investigations

During examination at the neurology outpatient clinic in early 2020, the patient appeared lethargic, unable to follow directions and demonstrated a loss of ability to speak or understand Dutch, consistent with akinetic mutism. A full cranial nerve examination was conducted, during which he exhibited repeated sleep episodes accompanied by myoclonic jerks. No facial asymmetry or abnormalities in tone, power, or sensation were observed. Reflexes were symmetric in all extremities and Babinski reflexes were absent. The gait was unsteady, but without ataxia. The patient had no mental capacity to perform higher-order cognitive tasks, such as the Mini-Mental State Examination (MMSE).

**Fig. 2:**
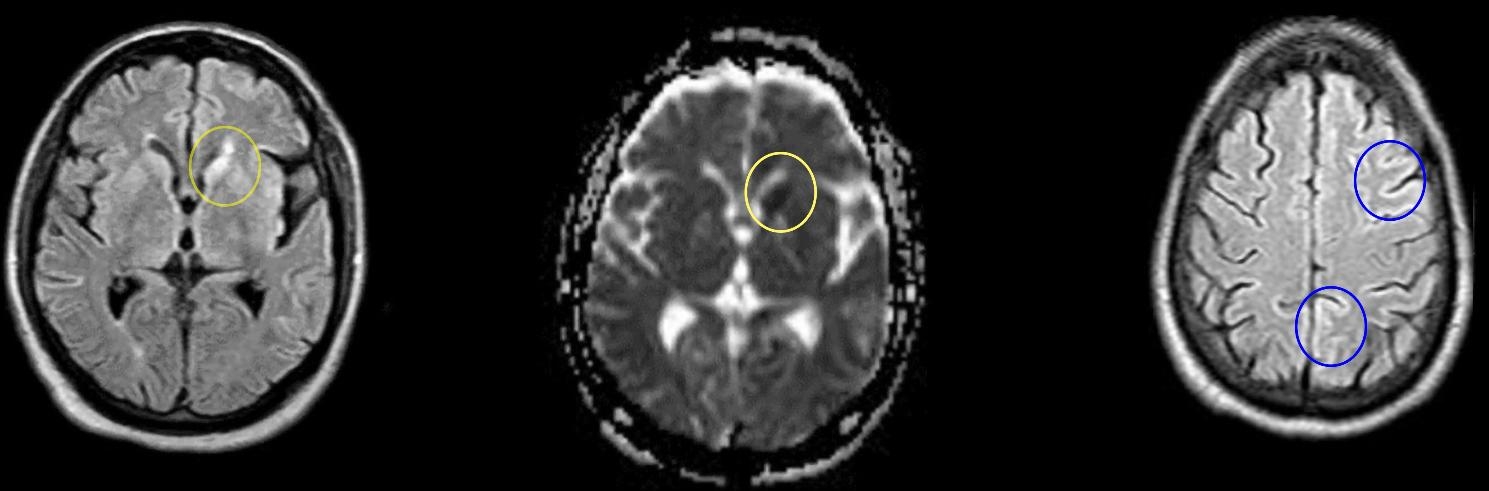
Brain MRI. The brain MRI revealed widespread atrophy, with both sides showing increased signal intensity in the caudate nucleus. The left caudate nucleus (marked yellow) was more prominently affected than the right, and there were similar changes observed in the thalamus on both sides. Additionally, signs of cortical ribboning were seen (marked blue), with the left side being more affected compared to the right

Laboratory tests, including basic blood count, non-fasting glucose level, liver function, renal function, electrolytes, and inflammatory markers were all within normal range (Table 1). Magnetic resonance imaging (MRI) showed ventricular widening due to generalized atrophy, along with hyperintensities at the caudate nucleus and thalami, which were more prominent on the left side compared to the right (Fig. 2). Fluid-attenuated inversion recovery (FLAIR) and diffusion weighted imaging (DWI) revealed cortical hyperintensities on the left side, suggestive of CJD. However, the MRI quality was compromised due to the patient’s movement, rendering it unreliable. No pleiocytosis was observed in the cerebrospinal fluid and both protein and glucose levels were normal (Table 2). The 14-3-3 and RT-QuIC tests were both positive. It is worth noting that the RT-QuIC test has a much higher sensitivity and specificity to detect prion protein in the cerebrospinal fluid than the 14-3-3 test [9]. The PRNP gene was sequenced, revealing heterozygosity at codon 129 (methionine/valine), but no mutations were found. The electroencephalogram (EEG) indicated diffuse slowing of background activity with paradoxical alpha waves (8–12 Hz) during blinking, but without triphasic wave complexes or epileptiform activity.

**Table 1:** Blood work-up at clinical presentation

	Result	Reference range
Hemoglobin	8.9 mmol/l	8.5–11.0 mmol/l
Hematocrit	0.43 l/l	0.40–0.50 l/l
Mean corpuscular volume	87.5 fl.	80.0–100.0 fl.
Leucocytes	10.5 × 10^9^/l	4.0–10.0 × 10^9^/l
Thrombocytes	304 × 10^9^/l	150–400 × 10^9^/l
White blood cell differentiation	Normal	Normal
Sodium	139 mmol/l	135–147 mmol/l
Potassium	4.0 mmol/l	3.5-5.0 mmol/l
eGFR (CKD-EPI)	71 kl/1.73 m [2]	< 60 kl/1.73 m [2]
Calcium	2.37 mmol/l	2.15–2.55 mmol/l
Albumin	39 g/l	35–52 g/l
Alkaline phosphatase	45 IU/l	< 115 IU/l
Alanine aminotransferase	36 IU/l	< 45 IU/l
Gamma-GT	31 IU/l	< 55 IU/l
Bilirubin	6 µmol/l	< 17 µmol/l
Glucose	13.9 mmol/l	<15 mmol/l
CRP	18 mg/l	< 10 mg/l

Abbreviations: eGFR = estimated glomerular filtration rate, using formula of Chronic Kidney Disease Epidemiology Collaboration (CKD-EPI), Gamma-GT = gamma glutamyl transpeptidase, CRP = C-reactive protein.

**Fig. 3:**
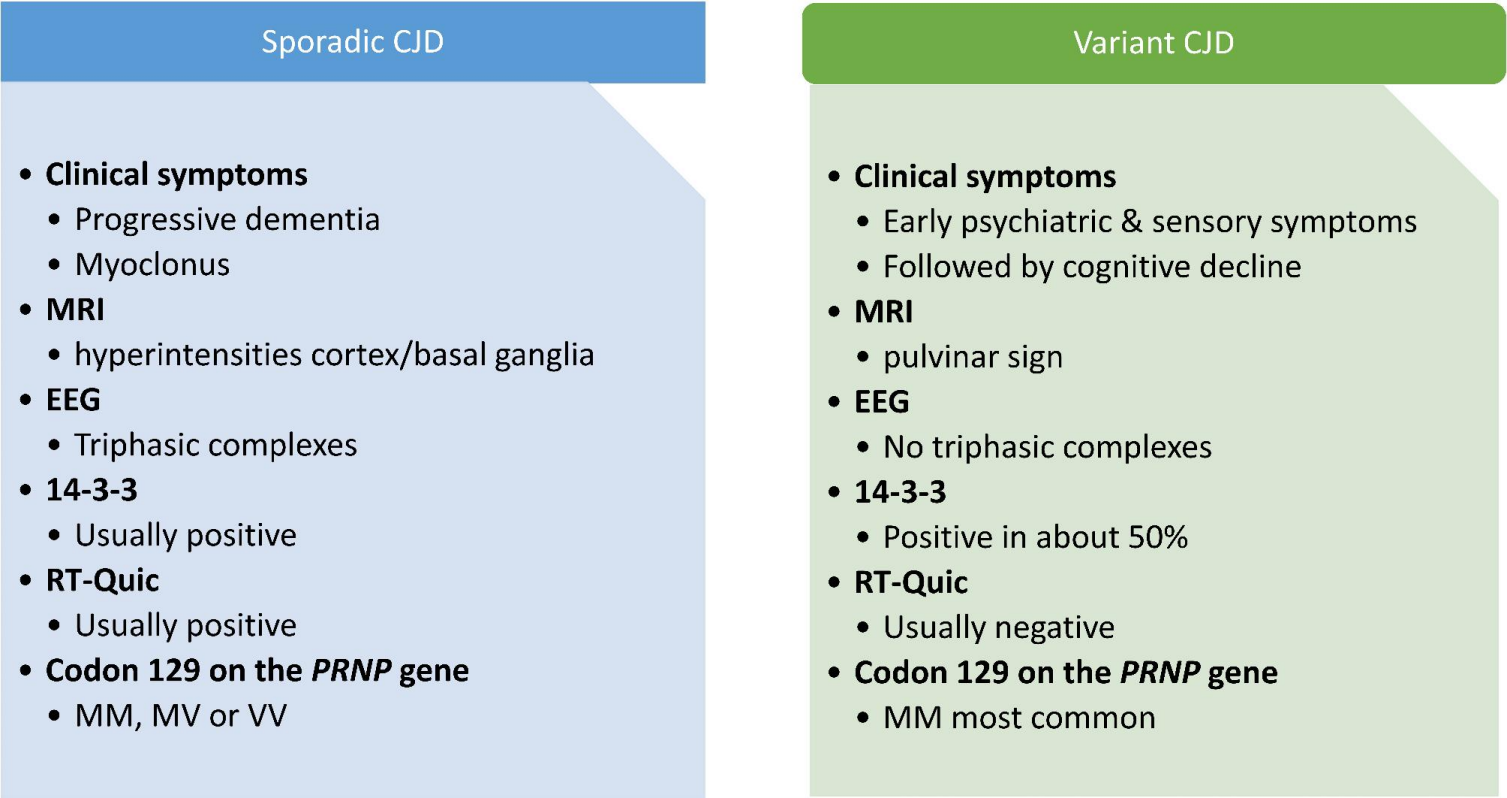
Clinical features of sporadic and variant CJD. Clinical features of sporadic and variant CJD – similarities and differences

**Table 2:** Results of cerebrospinal fluid analysis

	Result	Reference range
Erythrocytes	< 1 cell/µl	<= 1 cell/µl
Leucocytes	1 cell/µl	< 4 cell/µl
Protein	0.33 g/l	0.21–0.63 g/l
Glucose	5.4 mmol/l	2.0–6.5 mmol/L
14-3-3	positive	negative
RT-Quic test	positive	negative

Abbreviations: RT-Quic test = real-time quaking-induced conversion test

### Differential diagnosis

For at least a year (end of 2018–2019), the neuropsychiatric symptoms and allodynia were considered to be part of a persistent bereavement disorder alongside an aggravating CSS neuropathy, which had been treated by the general practitioner without much success. The patient’s deteriorating symptoms, including short- and long-term memory problems, prompted referral to a neurologist for suspected dementia in early 2020. The differential diagnosis at the time included frontotemporal dementia, Alzheimer’s disease, psychiatric disorders, vascular dementia, and inflammatory/metabolic disorders. However, none of these conditions could be confirmed based on the clinical features and investigations. CJD was suspected once the patient developed apraxia, myoclonus, and akinetic mutism in the following months, and was deemed probable once hyperintensities were found on MRI and both 14-3-3 and RT-QuIC test were positive.

After settling upon the diagnosis of CJD, determining the subtype was another challenge. We learned that the patient owned a catering company for many years, where he prepared and consumed beef amongst other products. Consistent with vCJD, the duration of illness was longer than 6 months, and both neuropsychiatric symptoms and persistent painful sensory symptoms (allodynia) were present early in the disease course. Nevertheless, the patient’s age, chronological order of symptoms, MRI (no evident bilateral pulvinar signs), positive RT-QuIC test and heterozygosity at codon 129 were more consistent with sCJD than vCJD (Fig. 3). Iatrogenic and familial CJD were unlikely, given the absence of invasive procedures or mutations on the *PRNP* gene. Considering all of the above, a clinical diagnosis of probable sCJD was made [10].

### Outcome and follow-up

Two months after establishing the diagnosis, the patient’s condition deteriorated rapidly and after being admitted to a nursing home, he passed away due to aspiration pneumonia. A brain autopsy for diagnostic confirmation was discussed with the family, but no permission was obtained.

## Discussion

This patient’s case illustrates the diverse clinical presentation of CJD in the context of multimorbidity, that resulted in considerable diagnostic delay. Besides coincidence, several mechanisms could underlie such an atypical presentation of CJD.

CSS is recognized as a systemic vasculitis affecting small-to-medium-sized vessels, that often extends to the nervous system [11]. Central nervous system vasculitis, as well as peripheral neuropathy, emerged among the top 10 misdiagnoses in a large study consisting of histopathologically confirmed sCJD cases [12]. This highlights the necessity of a thorough diagnostic workup when a patient with rare multimorbidity presents with clinical symptoms that may fulfill criteria for more than 1 disease. For instance, the allodynia of this patient was initially linked to peripheral neuropathy, which is a common neurologic manifestation of CSS. However, about 40% of individuals with the sporadic form of CJD show depositions of PrP^Sc^ in the peripheral nervous system, which can also elicit a neuropathy [13].  A worsening allodynia during the early disease course of CJD, furthermore complies with the criterium of painful sensory symptoms required for the variant form of CJD, [14] but is atypical for the sporadic form. On the other hand, behavioral changes that had been linked to a persistent bereavement disorder can well be neuropsychiatric manifestations of either vCJD or sCJD [15]. Physicians relying on diagnostic criteria may therefore not properly include CJD in the differential diagnosis, or may experience difficulty in distinguishing the subtype, when a patient is affected by multiple conditions.

Another uncommon occurrence was the stuttering that the patient presented with, which is a focal clinical symptom. About 1% of CJD cases present with an isolated language disorder as the primary clinical symptom [16]. Although lateralization for language was one of the earliest observations of brain asymmetry in humans, computed tomography studies have shown that petalia, referring to the protrusion of one cerebral hemisphere relative to the other, are among the most prominent brain asymmetries [17]. In individuals with situs solitus, this includes a combination of a right frontal lobe petalia and a left occipital lobe petalia. Interestingly, individuals with situs inversus usually retain lateralization of language to the left hemisphere, but exhibit a reversal in these petalia [18]. Both reversed petalia and reduced petalia result in an aberrant cerebral architecture and have been linked to stuttering [19]. While speech disturbances were not reported during the patients childhood, it is possible that the spongiform encephalopathy served as a trigger. Research on asymmetry in visceral organs and its association with brain function, [20], as well as studies focusing on brain asymmetry, [21] are still emerging and may provide additional insights on why the language circuit may be particularly vulnerable. Thus far, the evidence that genes controlling left/right visceral asymmetry are involved in neurological disorders predominantly stems from studies on handedness [22].

With only 1 to 2 cases diagnosed per million individuals, CJD is an exceptionally rare neurodegenerative disorder. The prevalence of CSS is 1 in 70,000-100,000 individuals and only 1 in 10,000 individuals have situs inversus totalis. Assuming complete independence, the probability of encountering a similar case as the one described is the multiplication of these separate probabilities, namely 1,43 × 10^− 15^. Such small probability would likely make this case a unique occurrence in human history. While the case presents unique challenges to reaching the CJD diagnosis, it also highlights the importance of a comprehensive workup amidst rare multimorbidity. Another hypothesis on the co-occurrence of these conditions revolves around the notion that PrP^c^ is involved in a wide variety of functions in the human body, [23] including: the regulation of the immune response by activating T-cells, neuronal differentiation, signal transduction and synaptic transmission, as well as both pro-apoptotic or anti-apoptotic properties (protection against anti-oxidative stress). Due to these pleiotropic effects of PrP^c^, the search for common causal mechanisms between CJD, situs inversus totalis as well as CSS remains a challenging endeavor.

## Conclusion

In this case-report, we describe the intricacies of diagnosing CJD, when the clinical presentation is obscured by rare multimorbidity. We highlight the importance of a comprehensive work-up for determining the CJD type, which is particularly important for variant CJD. Although the concurrent presence of multiple rare disorders in one patient is statistically unique and could well be a chance finding, several common underlying mechanisms are proposed, that can be explored in future studies.

### Electronic supplementary material

Below is the link to the electronic supplementary material.

Supplementary Material 1

Supplementary Material 2

## Data Availability

The medical record of the patient contains all the necessary data that was used for this case report. Given the privacy concerns, regulations and informed consent provided by the patient and their guardian, this record cannot be made publicly available. Further information can be obtained upon contacting the corresponding author.

## Abbreviations

CJD: Creutzfeldt-Jakob disease
PRNP: a protein coding gene
PrP: prion protein
sCJD: sporadic form of Creutzfeldt-Jakob disease
vCJD: variant form of Creutzfeldt-Jakob disease
MRI: magnetic resonance imaging
FLAIR: Fluid-attenuated inversion recovery
DWI: Diffusion-weighted imaging
RT-QuIC: real-time quaking-induced conversion test
MMSE: Mini-Mental State Examination
