# Supplementary material for: Diagnostic challenge of Creutzfeldt-Jakob disease in a patient with multimorbidity: a case-report
Source: BMC Neurol. 2023 Oct 2;23:346. doi: 10.1186/s12883-023-03401-5 (PMC10544493; doi:10.1186/s12883-023-03401-5)
Supplement: Supplementary file 2 — Supplementary Material 2 [file 12883_2023_3401_MOESM2_ESM.pdf]

Supplementary file 1

Diagnosis of Churg Strauss syndrome

Churg Strauss syndrome (CSS), also known as Eosinophilic granulomatosis with polyangiitis (EGPA), is a clinical syndrome for which the American College of Rheumatology (ACR) established six criteria in 1990 for a patient with documented vasculitis.[1] These criteria include asthma, mononeuritis multiplex, migratory or transient pulmonary opacities detected on radiography, paranasal sinus abnormality, and biopsy containing a blood vessel showing the accumulation of eosinophils in extravascular areas. If four or more of these criteria are met, a patient with vasculitis can be classified as having CSS with a sensitivity of 85% and a specificity of 99.7%.[2] The diagnosis of CSS for this patient dates back to 1998 when he exhibited asthma, peripheral blood eosinophilia, systemic vasculitis of the medium and small vessels, glomerulonephritis, and mononeuritis multiplex, which were documented in medical reports provided by the internist and the neurologist. Routine follow-up at the outpatient clinic included assessment of multiple organs commonly affected by CSS, such as radiologic examinations (**Figure 1-A**), kidney function assessment and a biopsy, as well as an electromyogram for monitoring mononeuritis multiplex. The CT-thorax included in the case report (**Figure 1-B**) also showed some ground glass calcifications indicative of parenchymal opacification, a common occurrence in CSS. Furthermore, the patient underwent assessment of p-ANCA and c-ANCA antibody blood tests, which supported the diagnosis (**Supplementary file 1, Supplementary Table 1**). While this information was not attached to the main manuscript to avoid shifting the focus to CSS rather than CJD, we have provided it here upon request for further context to the case.

Medical Report, 1998

Department: Department of Nephrology

Laboratory Investigation: BSE 50 mm/1st hour, Hb 7.4 mmol/l, MCV 85 fl, leukocytes 22.3 x 10^9/l, with 62% neutrophils, 21% eosinophils, 14% lymphocytes; total eosinophils 4630 x 10^6/l; Platelets 492 x 10^9/l. APTT 23 sec, PTT 11.2 sec. Bleeding time 2.5 min. Sodium 140 mmol/l, potassium 3.4 mmol/l, creatinine 95 µmol/l, urea 12.0 mmol/l, calcium 2.26 mmol/l, albumin 32 g/l, phosphate 1.1 mmol/l, bilirubin 4 µmol/l, OT 15 U/l, PT 33 U/l, AF 50 U/l, gamma-GT 83 U/l, glucose 8.6 mmol/l, LDH 136 U/l, bicarbonate 27.6 mmol/l.

Laboratory investigation (continued): ANA negative, anti-double-stranded DNA negative, Atypical ANCA antibodies detected, titer 1:256, Elisa-Pr3 negative, Elisa-MPO positive, Waaler-Roosetest positive (200E/ml), latex fixation test positive (100 E/ml), Positive IgM rheumatoid factors (25 E/ml), vitamin B12 600 pmol/l, C1q 137 IU/ml, C3 1.9 g/l, C4 336 mg/l, HBsAG negative.

Laboratory investigation (bacteria and viruses): CMV IgM negative, IgG positive; Varicella-zoster virus IgM negative, IgG positive; EBV: IgM negative, IgG positive; CBV anti-EBNA: negative, to be repeated when appropriate; herpes simplex virus: IgM negative, IgG positive; anti-GBM: negative. Urine culture: no growth.

Kidney and bladder ultrasound: slightly thickened bladder wall, no further abnormalities.

EMG: axonal neuropathy of left peroneal nerve and to a lesser extent, of the left posterior tibial nerve. Could fit with a mononeuritis multiplex.

Accurate kidney function investigation (12 x iohexol, 131I hippurate): GFR 57 ml/min, ERPF 225 ml/min, filtration fraction 0.25.

Kidney biopsy: 20 glomeruli, of which glomeruli are entirely sclerosed. Approximately 70% show extracapillary proliferation with partly fibrous and partly fibrocellular crescents. Focally, there is also proliferation of mesangium and the endocapillary. Focally, there is also an increase in mesangial matrix. In the interstitium, we see a dense inflammatory infiltrate of lymphocytes and eosinophilic granulocytes. There is focal perivasculitis, no vasculitis. In a few glomeruli we find fibrinoid necrosis. Tubules show no abnormalities. Immunofluorescence: not specific.

Urinalysis: albumin +/-, leukocytes 0 - 3 per high-power field, erythrocytes 4 - 10 per high-power field. 24-hour urine: sodium 167 mmol, potassium 112 mmol, urea 661 mmol, creatinine 11.0 mmol, total protein 0.99 g. Calculated creatinine clearance: 85 ml/min.

Issues:

1. Renal function impairment, sediment abnormalities, proteinuria.
2. Anemia.
3. Elevated sedimentation rate.
4. Eosinophilia.
5. Hyperpathia/sensory disturbances in the feet.
6. Skin lesions on the feet.

7. General malaise, history of fever, and others.

Brief summary october 1998: Laboratory investigation revealed elevated BSE, leukocytosis, significant eosinophilia, and increased creatinine levels (131 µmol/l). ANCA was atypical, MPO was positive, and the urinalysis showed protein and erythrocyte cylinders. A skin biopsy of foot lesions displayed a perivascular diffuse inflammatory pattern with eosinophilia. The working diagnosis was Churg-Strauss syndrome (CSS) due to the suspicion of vasculitis. Treatment with oral prednisone 50 mg once daily was initiated.

Discussion: From the literature, it is evident that the patient indeed fulfills the criteria for Churg-Strauss syndrome, as initially suspected. The presence of recent-onset asthma without significant family history, eosinophilia, skin vasculitis, and mononeuritis multiplex support this diagnosis. The severe kidney involvement indicated by the renal biopsy results corroborates the suspicion of renal vasculitis. The patient responded to pulse methylprednisolone therapy and oral prednisone, along with cyclophosphamide, which was initiated due to the pain from mononeuritis multiplex.

Interim conclusion:

1. Churg-Strauss syndrome with recent-onset bronchial asthma, eosinophilia, severe glomerulonephritis with impaired renal function, proteinuria, sediment abnormalities, mononeuritis multiplex, skin vasculitis, fever, malaise, weight loss, and anemia.

Conclusion: Churg-Strauss syndrome with bronchial asthma, eosinophilia, skin vasculitis, glomerulonephritis, and mononeuritis multiplex. Treated with Endoxan 100 mg, methylprednisolone (MPNS), and prednisone. Complicated by liver enzyme disorders possibly due to Endoxan or a past gallstone.

Final conclusion: the clinical image fits well with Churg-Strauss Syndrome.

May 2000: Endoxan discontinued, switched to Imuran, in combination with prednisone 10mg.

After treatment, the ANCA antibody tests improved.

Laboratory report after treatment:
Supplementary Table 1

ANCA antibodies	Reference	18th of September 2019
MPO Antibodies [P-ANCA]	0 - 5 kU/L	0,3
PR3 Antibodies [C-ANCA]	0 - 3 kU/L	0,7

References:

1. Masi AT, Hunder GG, Lie JT, et al. The American College of Rheumatology 1990 criteria for the classification of Churg-Strauss syndrome (allergic granulomatosis and angiitis). Arthritis & Rheumatism 1990;33:1094-1100.

2. Emmi G, Bettiol A, Gelain E, et al. Evidence-Based Guideline for the diagnosis and management of eosinophilic granulomatosis with polyangiitis. Nature Reviews Rheumatology 2023;19:378-393.
